# Real-time reconfigurable metasurfaces enabling agile terahertz wave front manipulation

**DOI:** 10.1038/s41377-023-01331-y

**Published:** 2023-11-27

**Authors:** Huixian Zhou, Cheng Zhang

**Affiliations:** School of Optical and Electronic Information & Wuhan National Laboratory for Optoelectronics, Huazhong University of Science and Technology, Wuhan, 430074 China

**Keywords:** Integrated optics, Terahertz optics

## Abstract

Real-time controlled programmable metasurfaces, having an array-of-subarrays architecture under the control of one-bit digital coding sequence, are demonstrated for rapid and precise multifunctional Terahertz wave front engineering.

Metasurfaces, quasi-two-dimensional arrays of subwavelength electromagnetic (EM) structures, have garnered significant attention in recent years. They facilitate light matter interaction at the subwavelength scales and empower precise manipulation of fundamental optical properties including amplitude, phase, and polarization^1–4^. Conventional metasurfaces are static and lack post-production adaptability. In contrast, dynamic metasurfaces offer tunable EM responses through various external stimuli, such as electrical^5^, optical^6^, mechanical^7^, thermal^8^, and chemical^9^ inputs. Such capability places dynamic metasurfaces at the forefront of advanced research and technological innovation in the metasurface field.

Efficiently controlling and manipulating terahertz (THz) beams is essential for various applications including high-speed wireless communication, spectroscopic imaging, and biomedical sensing. This necessitates high-performance, rapidly-responsive dynamic THz beam shaping devices. In the THz frequency range, achieving adjustability poses unique challenges compared to lower frequencies. To date, researchers have exploited candidates such as liquid crystal (LC)^10^, two-dimensional material^11^, two-dimensional electron gas (2DEG)^12^, semiconductor^13^, and phase-change material^14^ to replace diode structures^15,16^ used in microwave tunable metasurfaces. Electrically controlled THz beam shaping has emerged as a promising solution, which offers faster response, increased precision, smaller footprint, and reduced power consumption. While previous studies^12,17,18^ have achieved modulation frequencies up to the gigahertz (GHz) range and angular scanning precision down to a few degrees, striking an ideal balance between the response speed and scanning precision remains a significant challenge.

In a recently published paper in Light: Science & Applications, a collaborative team led by Prof. Hongxin Zeng and Prof. Yaxin Zhang from University of Electronic Science and Technology of China, along with Prof. Daniel M. Mittleman from Brown University, presents a real-time controlled programmable metasurface that satisfies the requirements for fast and precise THz wavefront manipulation^19^. The proposed metasurface employs GaN/AlGaN high-electron mobility transistors (HEMTs) as active switching components. HEMTs offer several advantages, including a sizable dynamic carrier density range, high electron drift velocity, minimal parasitic capacitance, and low power dissipation. By integrating a HEMT as the individual meta-atom with an asymmetric resonant structure and manipulating carrier concentration in the 2DEG through applied bias, it becomes possible to switch the resonant delay of an incident THz wave by 180° with uniform amplitude. This innovative design enables the implementation of one-bit phase-shifting metasurfaces while mitigating parasitic capacitance issues seen in previous studies. Furthermore, it allows for precise positioning of individual meta-atom element with subwavelength spacing, eliminating the need for integrated amplification or phase-control circuitry.

Fig. 1a presents schematic of the reconfigurable THz metasurface, which leverages the x-axis as the axis of symmetry for independent control of upper and lower symmetric subarrays in the ±y direction. Such dual-region mirrored subarray configuration enhances beam control flexibility compared to the traditional fully column-controlled approach. To achieve quasi-continuous beam scanning, a fractional phase-coding approach is employed (Fig. 1b). The researchers successfully demonstrated wide-band beam scanning in a view field ranging from 20° to 60° over a 70 GHz bandwidth (0.33 THz - 0.4 THz). At the operating frequency of 0.34 THz, the angular scanning accuracy can reach up to 1° per step. In addition, they demonstrated the generation of multi-beam steering through dual-region coding (Fig. 1c) and convolutional coding (Fig. 1d), as well as diffuse scattering through aperiodic (Golay-Rudin-Shapiro) coding (Fig. 1e), with switching speeds reaching up to 100 MHz. Furthermore, real-time beam tracking at 0.34 THz was implemented to verify the metasurface-assisted point-to-point signal transmission in various directions.

**Fig. 1:**
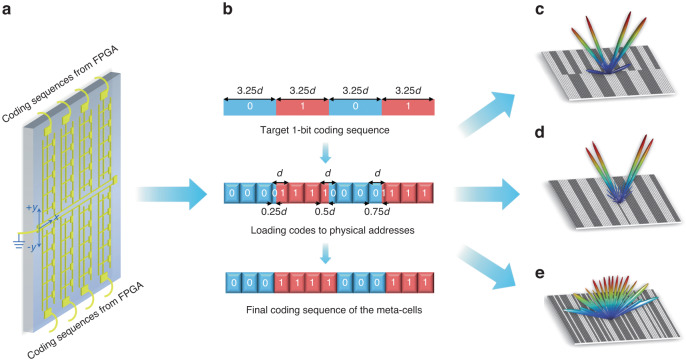
**Real-time programmable metasurface for multifunctional THz wave front engineering**. **a** 3D schematic of the real-time controlled THz programmable metasurface. **b** Schematic of the fractional phase-coding approach. **c**, **d** Multi-beam steering based on dual-region coding (**c**) and convolutional coding (**d**). **e** Diffuse scattering based on Golay-Rudin-Shapiro coding

This work presents an innovative approach towards achieving high-performance dynamic and intelligent THz metasurfaces, offering a balanced configuration that combines rapid response speed with great beam-steering precision. Looking ahead, there is a growing need for multi-bit coding methods to enhance the accuracy of beam steering and manipulation, necessitating the development of new switching mechanisms for meta-atoms and their associated device architectures. As dynamic metasurface technology advances, the aspiration is to achieve simultaneous and independent real-time control over multiple electromagnetic properties, including phase, amplitude, polarization, and more. Furthermore, achieving dynamic control through the combined use of multiple external stimuli (e.g., electrical, optical, mechanical means, etc.) could unlock new avenues and applications. However, these endeavors may introduce design and manufacturing complexities, as well as increased energy consumption. To tackle these challenges, optimization algorithms and neural networks could play a crucial role in streamlining the design and operation of multi-dimensional dynamic metasurfaces. Finally, dynamic metasurface technology is not limited to the THz and microwave regions, and can extend to other spectral domains such as the infrared and visible. However, these regions will require significantly different operational mechanisms and entirely new device architectures^20,21^. Achieving dynamic and intelligent metasurfaces for these spectral regions has the potential to address a notable challenge faced by traditional optical elements and, in turn, unlock new possibilities across various applications^22–26^, including smart imaging, adaptive optics, LiDAR, and advanced AR/VR displays.

## References

[1] Neshev D, Aharonovich I (2018). Optical metasurfaces: new generation building blocks for multi-functional optics. Light Sci. Appl..

[2] Zang XF (2021). Metasurfaces for manipulating terahertz waves. Light Adv. Manuf..

[3] Malek SC (2022). Multifunctional resonant wavefront-shaping meta-optics based on multilayer and multi-perturbation nonlocal metasurfaces. Light Sci. Appl..

[4] Zhang C (2020). Low-loss metasurface optics down to the deep ultraviolet region. Light Sci. Appl..

[5] Zeng BB (2018). Hybrid graphene metasurfaces for high-speed mid-infrared light modulation and single-pixel imaging. Light Sci. Appl..

[6] Liu K (2021). Active tuning of electromagnetically induced transparency from chalcogenide-only metasurface. Light Adv. Manuf..

[7] Zhao XG (2018). Electromechanically tunable metasurface transmission waveplate at terahertz frequencies. Optica.

[8] Chen HT (2010). Tuning the resonance in high-temperature superconducting terahertz metamaterials. Phys. Rev. Lett..

[9] Duan XY, Kamin S, Liu N (2017). Dynamic plasmonic colour display. Nat. Commun..

[10] Zhuang XL (2023). Active terahertz beam steering based on mechanical deformation of liquid crystal elastomer metasurface. Light Sci. Appl..

[11] Chen ZF (2018). Graphene controlled Brewster angle device for ultra broadband terahertz modulation. Nat. Commun..

[12] Zhang YX (2015). Gbps terahertz external modulator based on a composite metamaterial with a double-channel heterostructure. Nano Lett..

[13] Seren HR (2016). Nonlinear terahertz devices utilizing semiconducting plasmonic metamaterials. Light Sci. Appl..

[14] Chen BW (2022). Electrically addressable integrated intelligent terahertz metasurface. Sci. Adv..

[15] Bao L (2021). Programmable reflection-transmission shared-aperture metasurface for real-time control of electromagnetic waves in full space. Sci. Adv..

[16] Wang HL (2022). Broadband and programmable amplitude-phase-joint-coding information metasurface. ACS Appl. Mater. Interfaces.

[17] Venkatesh S (2020). A high-speed programmable and scalable terahertz holographic metasurface based on tiled CMOS chips. Nat. Electron..

[18] Monroe, N. M. et al. *Electronic THz pencil beam forming and 2D steering for high angular-resolution operation: a 98 × 98-unit 265GHz CMOS reflectarray with in-unit digital beam shaping and squint correction*. Proceedings of 2022 IEEE International Solid-State Circuits Conference (ISSCC). San Francisco: IEEE, 2022, 1–3.

[19] Lan F (2023). Real-time programmable metasurface for terahertz multifunctional wave front engineering. Light Sci. Appl..

[20] Abdelraouf OAM (2022). Recent advances in tunable metasurfaces: materials, design, and applications. ACS Nano.

[21] He Q, Sun SL, Zhou L (2019). Tunable/Reconfigurable metasurfaces: physics and applications. Research.

[22] Liu ZY (2023). Metasurface-enabled augmented reality display: a review. Adv. Photonics.

[23] Wang DY (2023). Structural color generation: from layered thin films to optical metasurfaces. Nanophotonics.

[24] Kim I (2021). Nanophotonics for light detection and ranging technology. Nat. Nanotechnol..

[25] Neshev DN, Miroshnichenko AE (2023). Enabling smart vision with metasurfaces. Nat. Photonics.

[26] Park JH, Lee B (2022). Holographic techniques for augmented reality and virtual reality near-eye displays. Light. Adv. Manuf..

